# Identification of a Hypoxia-Related lncRNA Biomarker Signature for Head and Neck Squamous Cell Carcinoma

**DOI:** 10.1155/2022/6775496

**Published:** 2022-01-19

**Authors:** Cuifang Yang, Xiang Zheng

**Affiliations:** Department of Otorhinolaryngology Head and Neck Surgery, Sir Run Run Hospital, Nanjing Medical University, Nanjing 211166, Jiangsu, China

## Abstract

**Purpose:**

Hypoxia is a leading hallmark of tumors, which is associated with carcinogenicity and dismal patient outcome. In this project, we tended to detect the prognostic value of hypoxic lncRNA and further generate a hypoxic lncRNA-based model in head and neck squamous cell carcinoma (HNSCC).

**Methods:**

We integrated the transcriptome and clinical information of HNSCC based on TCGA dataset. Univariate-multivariate Cox analysis was implemented to develop the signature according to hypoxia-related lncRNAs (HRlncRNAs) with greatly prognostic power in HNSCC. Next, the biomarker signature was tested using survival analysis and ROC plots. Moreover, we used GSEA to uncover the potential pathways of HRlncRNAs, and CIBERSORT and ssGSEA tools were applied to mirror the immune status of HNSCC patients.

**Results:**

Nine HRlncRNAs (LINC00460, AC144831.1, AC116914.2, MIAT, MSC-AS1, LINC01980, MYOSLID, AL357033.4, and LINC02195) were determined to develop a HRlncRNA-related signature (HRLS). High-HRLS group was associated with dismal patient outcome using survival analysis. Moreover, the HRLS was superior to classical clinical traits in forecasting survival rate of samples with HNSCC. GSEA unearthed the top six hallmarks in the HRLS-high group individuals. In addition, the HRLS was also bound up with the infiltration of macrophages, CD8 T cells, and activated mast cells.

**Conclusion:**

Our nominated nine-HRlncRNA risk model is robust and valuable tool for forecasting patient outcome in HNSCC.

## 1. Introduction

Head and neck squamous cell carcinomas (HNSCCs) are a heterogeneous group of neoplasms originating from the head and neck (HC) malignant areas, including the lip, oral cavity, nasopharynx, oropharynx, hypopharynx, and larynx [1]. HNSCC accounts for the majority of HC malignancies and has a high mortality rate. Over 700,000 people worldwide suffer from HNSCC each year, with a mortality rate of about 60% [2]. At present, clinical classification of HNSCC is generally based on anatomical site and tumor stage. HNSCC cases are usually diagnosed at advanced stage due to the shortage of the robust clinical screening indicators [3]. Although multitype treatments have been applied in HNSCC patients, the clinical outcome of HNSCC cases is still dismal [4].

Multiple studies have attempted to invest the molecular mechanism underlying its onset and progression and, by far, researchers have identified a subset of molecules as biomarkers in the detection of HNSCC. One intriguing report published in 2020 found that GATA3 could increase the risks for HNSCC by stabilizing HIF-1 and, recently, another group reported that UBE2C is closely bound up with the poor outcome of HNSCC [5, 6]. However, as a prognosis biomarker, single molecule can vary greatly and hinges on an individual's particular pathological status, making a comprehensive prognosis model based on multiple genes desperately needed.

Hypoxia is one of the hallmarks of the metabolic tumor microenvironment (TME) and occurs commonly in several solid tumors, such as liver cancer, stomach cancer, and breast carcinoma [7–10]. The development of hypoxic microenvironment is caused by overgrowth of the tumor and insufficient supply of oxygen in the blood [11]. Existing reports have suggested that hypoxia plays a central part in tumor aggressiveness and metastasis, resulting in drug resistance and treatment failure. Hypoxia also could boost cancer metastasis by activating cytokines related to tumor angiogenesis and invasion [12]. With the increase of tumor volume, the central region of HNSCC displays a high level of hypoxia [13]. However, the effective and reliable treatment for HNSCC tissue hypoxia has not been developed.

In the last decade, genomic and epigenetic studies have shown that long noncoding RNA (lncRNA), similar to protein-coding genes, exerts its crucial role in human cancer pathogenesis. Notably, greater than 50% of the human genome is in transcription as lncRNA which is a type of transcript of over 200 nucleotides [14]. lncRNA could mediate gene transcription and translation through epigenetic modifications or miRNA regulation. Under hypoxic status, lncRNAs can serve as oncogenes or tumor suppressors involved in regulation of carcinogenicity [15, 16]. For instance, lncRNA-BX111 induced by hypoxia could boost cell viability and metastasis by triggering ZEB1 transcription in pancreatic cancer [17]. In lung cancer, AC020978 has been proved to facilitate cell growth and activate glycolytic metabolism by promoting PKM2-induced HIF-1*α* upregulation after hypoxia treatment [18]. Unfortunately, the effect of hypoxia-related lncRNA (HRlncRNA) in HNSCC has been not comprehensively clarified yet.

In our project, we took advantage of the TCGA-HNSCC dataset and develop a risk signature based on hub HRlncRNAs which could offer reliable reference for patient outcome forecasting and individualized therapy of HNSCC patients.

## 2. Methods

### 2.1. Data Preparation

The HNSCC dataset with FPKM transcriptome data and basic clinical and survival information was collected from the Cancer Genome Atlas (TCGA) website. After filtering the samples with survival time <30 days, a total of 490 HNSCC cases were selected for the next analysis. We collected a list of 200 hypoxia-related genes (HRGs) from the MSigDB (Supplementary Table 1) [19].

### 2.2. Identification of Differentially Expressed HRlncRNAs

Then, we collected differentially expressed lncRNAs (DElncRNAs) using the Limma package in R project (|fold change (FC)| = 1.0 and *p* < 0.05). Moreover, correlation analysis was implemented to determine the HRlncRNAs based on the 200 HRGs in HNSCC and (|cor| > 0.3, *p* < 0.001) and differentially expressed HRlncRNAs were obtained by overlapping with DElncRNAs.

### 2.3. Construction of the Prognostic Signature

To develop a lncRNA-based signature, all HNSCC samples were equally and randomly divided into a discovery cohort and a validation cohort in a 1 : 1 ratio. Performing univariate Cox method, we identified underlying HRlncRNAs which display greatly prognostic value of HNSCC in the discovery cohort (*p* < 0.05). Subsequently, the candidate HRlncRNAs were analyzed by multivariate Cox regression to create a HRlncRNA signature (HRLS). The risk power of HRLS =  ∑exp(HRlncRNAs)*∗β*. The *β* is the coefficient of each candidate HRlncRNAs from multivariate Cox analysis.

### 2.4. Development of the Predictive Nomogram

A total of 490 cases comprised corresponding clinical data for the univariate and multivariate methods. To better develop the predictive ability of HRLS, we created a nomogram based on HRLS and other clinicopathological variables for outcome forecasting in HNSCC. Calibration curves were generated to verify the nomogram.

### 2.5. Gene Set Enrichment Analysis

To determine the valuable function and mechanism-related HRLS, we performed GSEA analysis on the basis of the hallmark gene sets. This project implemented GSEA method to identify those enrichment terms in HRLS-high group and the gene sets were collected from the MSigDB. The number of random combinations of genomes per analysis was set at 1,000. *p* < 0.05 was considered as statistically significant.

### 2.6. Analysis of Tumor-Infiltrating Immunocyte

The immune landscape of HNSCC samples was characterized by CIBERSORT which is an immune-related algorithm for analyzing the abundance of 22 immunocyte types. In addition, single-sample gene set enrichment analysis (ssGSEA) was employed to estimate the immune function between two subgroups.

### 2.7. Statistical Analysis

All statistical data were analyzed by R version 4.0.5. Kaplan–Meier (KM) survival was instrumental in detecting survival distinctions between two HRLS groups. The specificity and reliability of the HRLS were confirmed using ROC curves.

## 3. Results

### 3.1. Identification of Differentially Expressed HRlncRNAs

**Figure 1:**
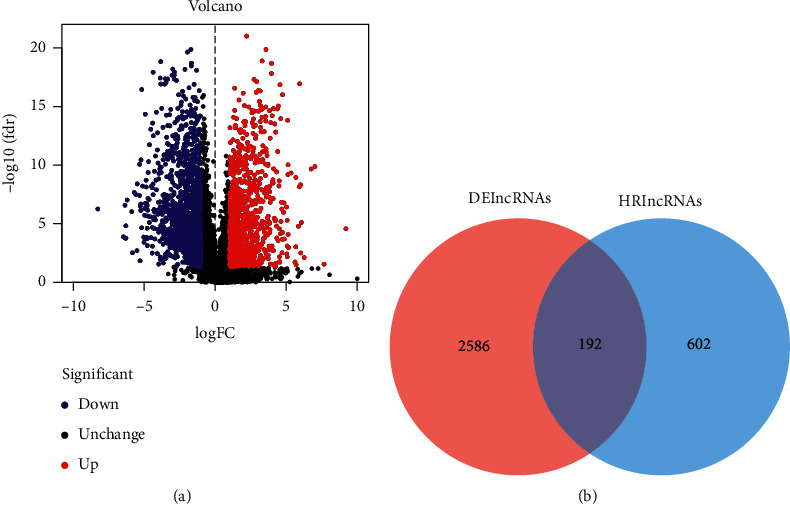
Differentially expressed hypoxia-related lncRNA (DEHRlncRNAs). (a) Volcano plot of DElncRNAs in TCGA-HNSCC dataset. (b) The Venn plot of lncRNA among DElncRNAs and HRlncRNAs.

We first unearthed 2778 DElncRNAs using differential expression analysis (991 upregulated and 1787 downregulated; Figure 1(a)). Then, a total of 794 HRlncRNAs were uncovered by performing correlation analysis based on the hypoxic gene sets. We collected 192 differentially expressed HRlncRNAs which overlapped with DElncRNA sets and HRlncRNA sets (Figure 1(b)).

### 3.2. Construction of the HRLS

**Figure 2:**
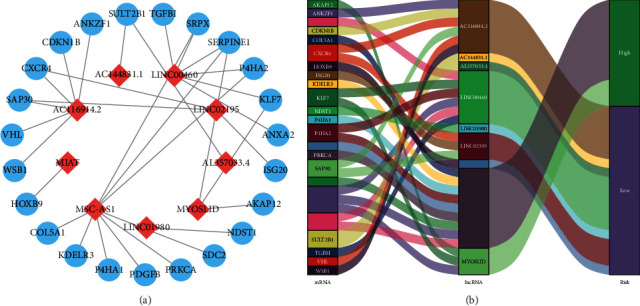
The relationship between model lncRNA and corresponding mRNA. (a) Model lncRNA-mRNA coexpression network. (b) Sankey plot showed the correlation of signature lncRNA-mRNA.

We integrated survival information from the HNSCC cohort in TCGA and removed cases with survival time <30 days. Next, 246 patients were randomly assigned into the discovery cohort. Conducting univariable Cox analysis, we identified 40 prognostic-associated HRlncRNAs in the discovery set. Then, all these HRlncRNAs were analyzed by a multivariate analysis. Ultimately, nine HRlncRNAs (AC116914.2, AC144831.1, AL357033.4, LINC00460, LINC01980, LINC02195, MIAT, MSC-AS1, and MYOSLID) were screened to create the HRLS (Table 1). The risk score = [AC116914.2 × (−0.0346)] + [AC144831.1  ×  (−0.1636)] + [AL357033.4 × (−0.2128)] + [LINC00460  ×  (−0.2781)] + [LINC01980 × (−0.0072)] +  [LINC02195  ×  (−0.0610)]  +  [MIAT  ×  (−0.3569)]  +  [MSC-AS1 × (0.0804)] + [MYOSLID × (0.1368)]. The relationship between HRlncRNA and HRG is shown in Figures 2(a) and 2(b).

**Figure 3:**
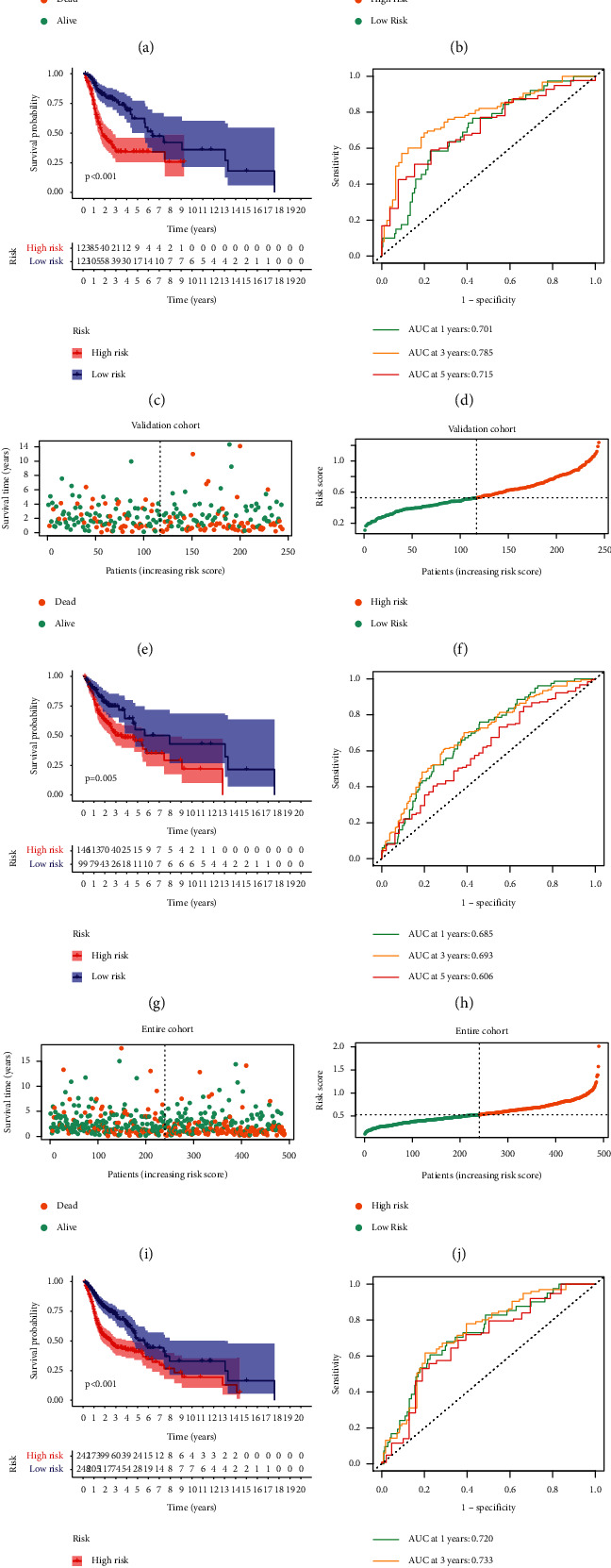
Predictive power of the HRLS. (a) The layout of risk score, (b) the survival status, (c) survival curves, (d) and ROC curves of the HRLS in the discovery cohort. (e–l) The verification sets using test and entire cohorts.

All patients were classified into high- and low-risk groups based on the median value of risk score. As shown in (Figures 3(a) and 3(b)), our established HRLS displayed favorable performance in the discovery cohort. KM survival curves revealed that HRLS-high group had dismal patient outcome, but HRLS-low group presented better patient outcome (Figure 3(c)). In addition, the results of ROC curves suggested that AUC values were 0.701, 0.785, and 0.715 for 1-, 3-, and 5-year survival, respectively (Figure 3(d)). At the same time, the above analyses were also applied to detect the performance of the HRLS using verification set. As we expected, the similar trend was observed in the two verification sets (Figure 3).

### 3.3. Verification of Nine Signature HRlncRNAs

**Figure 4:**
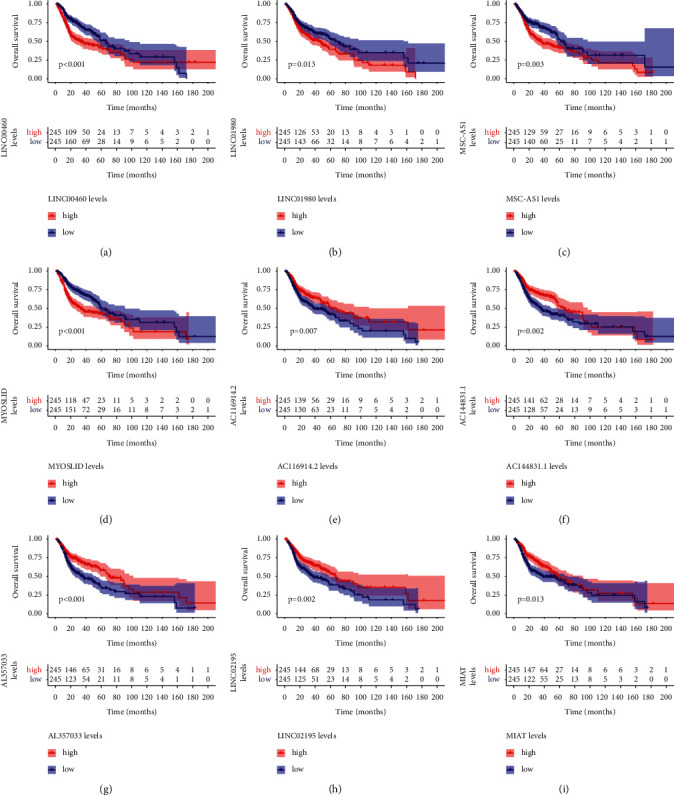
Prognostic values of the nine HRlncRNAs. (a) LINC00460. (b) LINC01980. (c) MSC−AS1. (d) MYOSLID. (e) AC116914.2. (f) AC144831.1. (g) AL357033.4. (h) LINC02195. (i) MIAT.

Then, we determined the prognostic association of nine hub lncRNAs. The results showed that dismal survival rates were uncovered in the high expression of LINC00460, LINC01980, MSC−AS1, and MYOSLID (Figures 4(a)–4(d)) and the low expression of AC116914.2, AC144831.1, AL357033.4, LINC02195, and MIAT (Figures 4(e)–4(i)).

### 3.4. Subgroup Analysis of the HRLS

**Figure 5:**
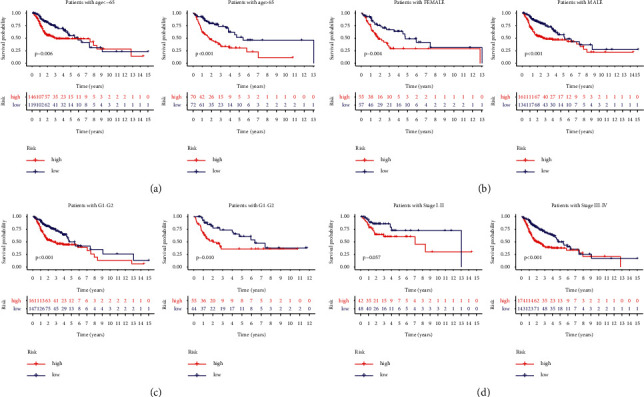
Subgroup analysis of the HRLS for HNSCC samples. (a) Age. (b) Gender. (c) Grade. (d) Stage.

Also, we confirmed the prognostic power of the HRLS in terms of the subgroup of HNSCC cohort. The entire HNSCC cohort were classified into several subgroups, including age subgroup, gender subgroup, grade subgroup, and stage subgroup. The outcomes of patients in HRLS-high group were favorable according to the abovementioned different subgroups (Figure 5).

### 3.5. Development of a Nomogram

**Figure 6:**
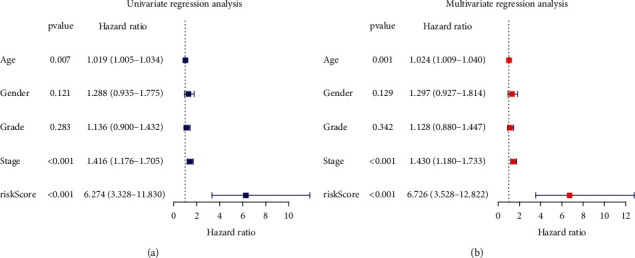
Analysis of the independence of the HRLS. (a) Univariate regression analysis. (b) Multivariate regression analysis.

**Figure 7:**
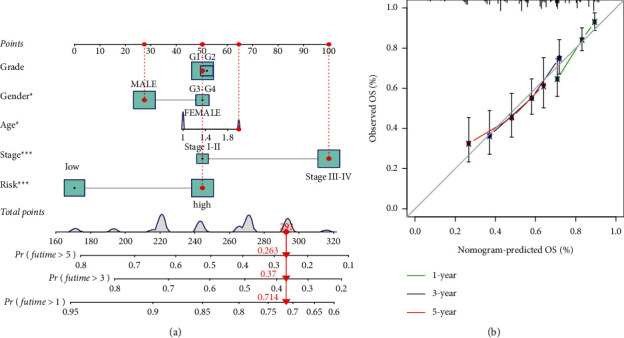
Establishment of the prognostic nomogram. (a) Nomogram to forecast patient's outcome in 1 , 3, or 5 years. (b) The 1-, 3-, or 5-year calibration plots for nomogram.

Cox stepwise regression of the HNSCC set indicated the independence of the HRLS in forecasting survival of patients. Both univariate and multivariate methods showed that risk score was meaningful for forecasting clinical outcome (Figures 6(a) and 6(b)). To further enlarge the predictive value of the HRLS, we adopted risk score to generate a nomogram (Figure 7(a)). In addition, calibration curves show a favorable adaptation of our proposed nomogram for predicting survival (Figure 7(b)).

### 3.6. Immune Status between Two HRLS Groups

**Figure 8:**
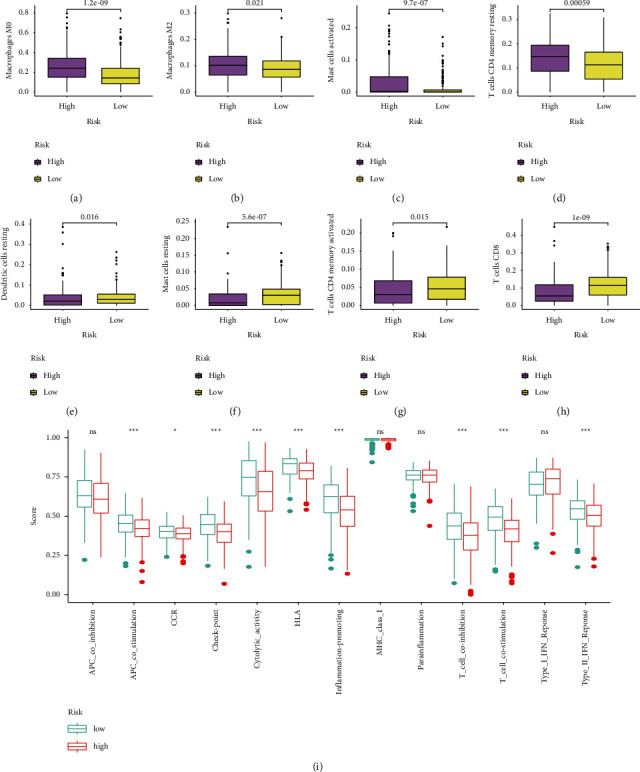
Immune landscape of the HRLS. (a) Macrophages M0. (b) Macrophages M2. (c) Activated mast cells. (d) Resting T cells CD4 memory. (e) Resting dendritic cells. (f) Resting mast cells. (g) Activated T cells CD4 memory. (h) T cells CD8. (i) Immune-related function analysis of the HRLS.

We further assessed the differences in the immune status based on immunocyte infiltration between two subgroups. HRLS-high group displayed remarkably higher abundances of macrophages M0, macrophages M2, activated mast cells, and resting T cells CD4 memory (Figures 8(a)–8(d)), whereas extremely lower abundances of resting dendritic cells, resting mast cells, activated T cells CD4 memory, and T cells CD8 (Figures 8(e)–8(h)). Furthermore, ssGSEA showed that APC costimulation, check point, cytolytic, HLA, inflammation-promoting, and type II INF responses were enriched in low-risk cohort (Figure 8(i)).

### 3.7. Correlation between m6A-Related Markers and Risk Score

**Figure 9:**
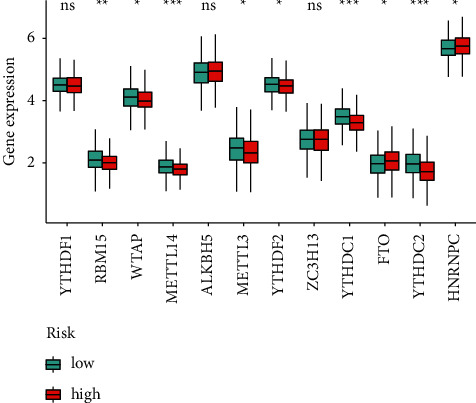
Association between m6A-related genes and the HRLS.

Given the importance of m6A-related genes in tumor regulation, we found that the expressions of RBM15, WTAP, METTL14, METTL3, YTHDF2, YTHDC1, YTHDC2, FTO, and HNRNPC were dramatically different between the two groups (Figure 9).

### 3.8. GSEA Enrichment of the HRLS

**Figure 10:**
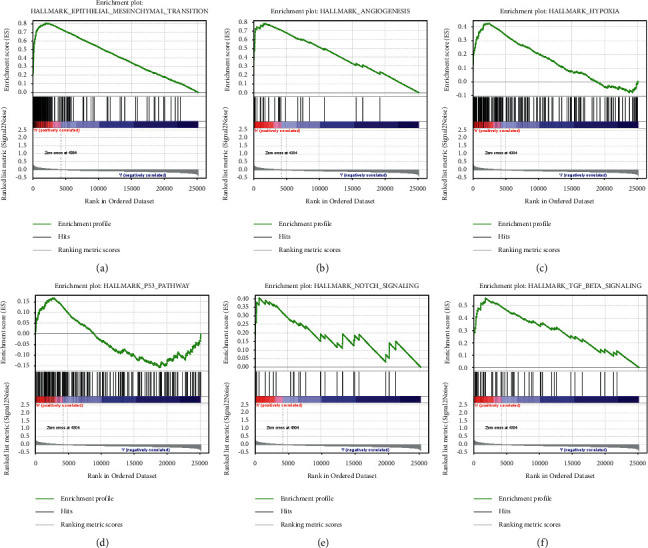
GESA of the HRLS. (a) Epithelial-mesenchymal transition. (b) Angiogenesis. (c) Hypoxia. (d) p53 pathway. (e) NOTCH signaling. (f) TNF-beta signaling.

GSEA displayed that there were six top hallmarks activated in the group with high-risk status, including “epithelial-mesenchymal transition,” “angiogenesis,” “hypoxia,” “p53 pathway,” “NOTCH signaling,” and “TNF-beta signaling” (Figure 10).

## 4. Discussion

HNSCC is a common head and neck cancer with highly heterogeneous nature, which has high mortality [1]. Hypoxia, a characteristic hallmark of several malignancies, is the pivotal cause of tumor progression and treatment resistance [13]. Currently, lncRNA-based risk model has increasingly gained interest as a result of its superior predictive capability [20]. However, prognostic model based on hypoxic lncRNAs has yet to be comprehensively analyzed.

In our project, we first collected 192 differentially expressed HRlncRNAs based on Pearson correlation analysis and differential analysis. Next, we focused on these lncRNAs with prognostic power. In the discovery set, nine HRlncRNAs were screened to construct the HRLS via Cox relevant analysis. Survival curves and ROC analyses illustrated the robust perform of the HRLS for forecasting patient outcome. Furthermore, the HRLS was proved to be an independent indicator for clinical outcome. In addition, we explored the potency of the HRLS in immune activity. The results showed that the risk scores were closely bound up with the abundance of TAM, activated mast cells, and CD8 cells, which could offer valuable reference for individual immunotherapy.

Our nominated HRLS is constituted by nine HRlncRNAs which were greatly associated with outcomes of HNSCC cases. Among these nine HRlncRNAs, MSC-AS1 and MYOSLID are underlying hazardous factors, but LINC00460, AC144831.1, AC116914.2, MIAT, LINC01980 AL357033.4, and LINC02195 underlying favorable indicators. After searching the available reports, we observed that LINC00460 AC116914.2, MIAT, MSC-AS1, LINC01980, MYOSLID, and LINC02195 are proven to be distinctly associated with various tumors.

Existing literatures show that LINC00460 exerts its role in the aggressiveness of several cancers. Jiang et al. revealed the high expression value of LINC00460 in HNSCC cell lines and uncovered that knockdown LINC00460 could inhibit cell viability and metastasis by promoting PRDX1 into the nucleus [21]. In colorectal cancer (CRC), LINC00460 was shown to serve as an oncogene of CRC that got involved in carcinogenesis by heightening the stabilization of HMGB1 at mRNA level [22]. As suggested by Zhou et al., MIAT could exert oncogenic role in regulation of cell growth and EMT through binding with miR-150-5p, providing a novel insight for the management of ovarian cancer [23]. Yao et al. demonstrated that high expression MSC-AS1 could facilitate the malignant behavior of nasopharyngeal cancer via miR-524-5p/NR4A2 axis [24]. Zhang et al. reported that MSC-AS1 was associated with aggressiveness and cisplatin sensitivity in osteosarcoma [25]. The LINC01980 is a new biomarker studied in digestive system tumors, such as esophageal squamous cell carcinoma (ESCC) and liver cancer (LC). Liang found that LINC01980 could aggravate cell viability and migration in a ceRNA-dependent way, indicating that it might serve as a possible marker for patient outcome of ESCC [26]. Additionally, LINC01980 also triggers the development of LC by targeting caspase 9 [27].

MYOSLID was confirmed as a slug-associated lncRNA involved in aggravation of cell invasion and metastasis in HNSCC [28]. Han et al. interrogated the functional effect in gastric carcinoma and they indicated that downregulation of MYOSLID markedly blocked cancer cell growth and induced apoptosis. In regard to the molecular mechanism, MYOSLID acts as a ceRNA targeting MCL-1 by binding with miR-29c-3p [29]. Li et al.'s study reveals that LINC02195 was tightly bound up with MHC I protein in HNSCC cells and could function as valuable prognostic indicator for HNSCC patients [30]. In addition, AC116914.2 has been determined as an autophagic lncRNA to set up a prognostic model in HNSCC [31]. AC144831.1 and AL357033.4 have not been previously reported in cancers.

In recent years, tumor microenvironment (TME) has been a hot spot in oncology research and cancer therapy. It is identified as an intricate cellular environment with the presence of tumor cells, various immunocytes, and stromal cells [32]. Among these tumor-infiltrating immunocytes, TAM is the predominant immune component in TME. TAM is recognized crucial factor in tumor development and is bound up with tumor growth, migration, and therapy failure [33]. As we all known, TAM can be transformed into two phenotypes: M1 polarization and M2 polarization. Research showed that M1 polarization might be involved in antitumor process and proinflammatory regulation. M2 polarization can motivate tumor proliferation and induce immunosuppressive effects in TME [34, 35]. Moreover, M2-like macrophage considered valuable indicator which could forecast the poor prognosis of patients with HNSCC [32]. Our analysis revealed that M0 macrophage and M2 macrophage had positive relevance to high risk, suggesting that dismal prognosis of HRLS-high group might be associated with higher M2 macrophage infiltration. In our project, HRLS-low group presented higher proportions of CD8 T cells and activated mast cells. CD8 T cell, important player in cancer management, is essential in human body defense against tumor [36]. Also, the infiltration level of CD8 T cell is closely related to patient outcome of HNSCC cases [37]. In addition, activated mast cells could release tissue kallikrein (TK1), which in turn boosts the viability and invasion of tumor cells through upregulation of angio-permeability [38].

GSEA presented that the HRLS-high groups were concentrated on the hallmarks including “epithelial-mesenchymal transition,” “angiogenesis,” “hypoxia,” “p53 pathway,” “NOTCH signaling,” and “TNF-beta signaling.” Epithelial-mesenchymal transition (EMT) is a cellular procedure in which cells undergo a transition between epithelial and mesenchymal phenotypes, characterized by changes in the expression of EMT-related markers [39]. Research proved that EMT pathway plays a central part in tumor migration and invasion. For example, NGF/TrkA axis could reduce the chemotherapy sensitivity through EMT signaling in HNSCC [40]. In addition, TRAF6 is closely related to EMT pathway and cancer cell stemness, which mediate migration and invasion of HNSCC cells [41]. Consistent with the predictions of our model, hypoxia was notably enriched in the HRLS-high group. Hypoxia, a frequent condition in HNSCC, has been reported to be a critical component of malignant behavior and treatment resistance [42]. Xu et al. demonstrated that MFAP5 could boost EMT process by activation of AKT under hypoxia status [43]. After hypoxia treatment in HNSCC cell lines, the expression of SRGN was increased by secretion of cancer-associated fibroblasts, which in turn stimulates tumor exacerbation via Wnt/*β*-catenin axis [44]. Moreover, p53 is a classical tumor suppressor biomarker in different cancers. NR5A2, a novel target in cancer management, was proved to be involved in regulation of HNSCC cell viability through p53-dependent way [45]. Study showed that NOTCH pathway plays a dual role in carcinogenesis of HNSCC. NOTCH1, for example, could function as a suppressor in HNSCC in an inactivating mutations manner. On the other hand, NOTCH1 also might exacerbate cell proliferation and progression of HNSCC through activating mutations [46].

Nevertheless, several parts of the present project warrant improvement. Firstly, both external cohort and prospective research are required to confirm the performance of our proposed HRLS. In addition, we need to further uncover the concrete mechanisms of HRLS through wet experiments.

In summary, we screened nine specific hypoxia-correlated lncRNAs which were included to develop a prognostic signature. The HRLS could forecast the prognosis and immune landscape of HNSCC cases, subsequently providing favorable therapeutic option for HNSCC patients.

## Figures and Tables

**Table 1:** Nine prognostic HRlncRNA greatly associated with prognosis of HNSCC cases.

Gene	Coefficient	Hazard ratio (95% CI)	*P* value
AC116914.2	−0.0346	0.61 (0.43–0.86)	0.005
AC144831.1	−0.1636	0.60 (0.44–0.81)	<0.001
AL357033.4	−0.2128	0.67 (0.54–0.84)	<0.001
LINC00460	−0.2781	1.32 (1.15–1.51)	<0.001
LINC01980	−0.0072	1.16 (1.03–1.30)	0.015
LINC02195	−0.0610	0.76 (0.62–0.94)	0.009
MIAT	−0.3569	0.74 (0.58–0.94)	0.013
MSC-AS1	0.0804	1.33 (1.10–1.62)	0.003
MYOSLID	0.1368	1.31 (1.15–1.49)	<0.001

## Data Availability

Public data were analyzed in this project. All data can be collected from TCGA (https://portal.gdc.cancer.gov/).

## Supplementary Materials

Supplementary Materials Supplementary Table 1: the list of hypoxia-related genes. .
